# Conceptual change of disaster management models: A thematic analysis

**DOI:** 10.4102/jamba.v10i1.451

**Published:** 2018-04-19

**Authors:** Mehdi Nojavan, Esmail Salehi, Babak Omidvar

**Affiliations:** 1 Department of Environmental Planning, University of Tehran, Iran; 2 Department of Environmental Engineering, University of Tehran, Iran

## Abstract

Different models have been proposed for disaster management by researchers and agencies. Despite their efficiency in some locations, disasters are still a fundamental challenge in the way of sustainable development. The purpose of this research is developing a comprehensive conceptual model for disaster management using thematic analysis. In this regard, first, disaster management models are collected. In the next stage, the themes of each model are extracted and categorised in three phases. In the first phase that is descriptive coding, available elements in each model are extracted as code and the basic themes are recognised. Then, in the phase of interpretive coding, basic themes are classified in three categories that are called organising themes (i.e. hazard assessment, risk management and management actions). In the final phase, strategic management is selected as the global or overarching theme to integrate all the other themes. Based on thematic analysis, it can be concluded that disaster management has three main elements that are the three organising themes. Therefore, comprehensive model of disaster management should include these three elements and their sub-basic themes that is called the ideal or criterion type. Results showed that some scientists have looked at disaster management one dimensionally (one theme). Even in two-dimensional models, one dimension has advantage over the other one. While the proposed typology in this study showed that the comprehensive model should include all the three mentioned elements.

## Introduction

One of the environmental difficulties and problems that the world’s biggest human settlements are facing is the problem of disasters. In recent decades, a striking world-wide trend towards rising fatalities and economic losses because of natural and man-made hazards can be seen all over the world. One of the important and influencing factors for this increment is growing urbanisation, and most importantly, the settlements that are particularly prone to supply crisis, social disorganisation, political unrest, natural and man-made disasters because of the high population density and extreme dynamics of development (Kraas 2008). Considering that countries all over the world are increasingly urbanising (Dutta 2012), according to the United Nations forecast, it is expected that almost 80% of the world will live in cities by 2050 (Jha, Miner & Geddes 2012). This means that urban areas will turn into the main location for many potential disasters (León & March 2014). Therefore, planning for disasters in urban areas should be considered as the main and basic strategy in all phases of urban planning.

Disaster management is considered as one of the main factors affecting disaster prevention and an effective strategy when they happen. However, despite the fact that disasters have always been along with humans, disaster management is still a relatively new profession and scientific field (Asgary 2006). As a new profession and scientific field, disaster management also needs to develop its principles and foundation to be able to continue as a professional and scientific field. In line with that, researchers and experts have always been trying to find principles for this field and profession. Kelly (1998) defined four main reasons for the necessity of developing a (theoretical) model for natural disasters management. He stated that a model can simplify complex events by helping to distinguish between critical elements.

In this regard, different models have been proposed for disaster management by researchers and agencies. Quarantelli (1994) attempted to develop the principles of disaster management. To him, ‘principles of good and efficient disaster planning’ have certain characteristics that can be used as general principles. Cuny (1998) defined a cycle for disaster management, which is one of the most complete cycles in which the managerial and executive measures and activities to be taken during a disaster have been considered. Mileti (1999) developed a set of principles for preventing and reducing the negative effects of natural disasters. Kimberly (2003) proposed a four-phase model of disaster management. In this model, special attention has been paid to emergency management. McEntire, Crocker and Peters (2010) proposed an integrated approach for modelling vulnerability based on physical science, engineering, structural and organisational schools. Organisational school, which is the most recent school in natural hazards, has been formed based on the concept of resiliency. Van der Waldt (2013) also proposed steps towards a unifying theory as overarching paradigm for disaster risk management.

However, the process of forming different models has been criticised step-step throughout history and new approaches have been formed and each of them has been criticised, considering the historic events. Alexander (1997) believes that approaches and models of disaster management and planning have not had any significant progress based on the following factors: death tolls have not fallen dramatically in response to improved mitigation, large-scale transfer of technology has not occurred and disaster relief has not been adequately combined with mitigation and economic development. Alexander (2016) also reviewed the modern-day challenges facing researchers, scholars and practitioners who work in the field of disaster risk reduction. He stated that there is a need for a major revision in the body of disaster theory so that it can take into account dynamic changes in the modern world. On the other hand, disaster theory must adapt to new conditions if it is to remain the ‘road map’ that clarifies complex realities and enables disasters to be managed. Asghar, Alahakoon and Churilov (2006) argue that suggested models and approaches so far have some limitations. For example, design of most of the models revolves around the four main phases of disaster management: prevention, mitigation, response and recovery. In other words, these models are not designed to cover all aspects of disaster management, such as hazard assessment, risk management and their sub-components. Also, there is no model or approach that can encapsulate main and major activities of disaster management within a framework.

According to Contreras (2016), a number of indices have been developed for measuring vulnerability to disasters, but little attention has been paid to recovery indices. In other research, Bendito and Barrios (2016) discussed that developing a trans-disciplinary strategy that effectively integrates disciplines, approaches and knowledge systems will lead to greater and more sustainable impacts, together with more efficient use of financial resources.

Finally, Scott et al. (2016) developed a unique monitoring and evaluating framework for use by disaster risk management programmes to track the outcomes of their interventions and ultimately raise standards in this area. In that study, they discussed and noted a weakness in relation to monitoring and evaluating of disaster risk management and highlighted that disaster risk management capacity development programmes typically need help to develop and implement robust monitoring and evaluating systems.

Briefly, a review over the literature of previous theories reveals a big gap in disaster management approaches, for designing and establishing efficient models to confront extensive and extreme disasters (Mohapatra 2009). Different researchers have considered disaster management as a multistage process. So, each model has been designed for a specific need in the field of disaster management and there have been no comprehensive research about the structure of these models, typology and classification before.

Considering the weaknesses of the previous models, despite their efficiency in some locations and under certain circumstances, disasters are still a fundamental challenge in the way of sustainable development. Therefore, disaster management requires an arranged and regular system with an appropriate approach and model so that it can largely reduce the possibility of crisis negative consequences.

The primary aim of this research is to assess the current models of disaster management through thematic analysis. The secondary aim of the article is to classify and recognise organising themes of disaster management. The third aim of this study is to present a typology and a comprehensive conceptual model for disaster management.

Through thematic analysis as research method, this article will analyse the content of disaster management models to ascertain the basic and organising themes of models in order to develop a comprehensive model. It should be noted that this analysis pertains to models that have been proposed in various countries.

## Research methodology

In order to develop a conceptual model for disaster management that can be used at different levels, combination of thematic analysis, classification and typology is used to overcome the conceptual complications and inconsistencies that exist among the models at first sight and to simplify development of the final model.

Thematic analysis is the approach of data analysis and reduction that is used to segment, categorise, summarise and reconstruct the qualitative data (Given 2008; Mills, Durepos & Wiebe 2010). Classification is also very essential and plays a fundamental role in social sciences. In the simplest form, classification means regular arranging of entities in groups or categories, based on their similarities to each other. In statistical sciences, the goal of classification is generally to minimise intra-group variances and maximise inter-group variances (Bailey 1994).

Typology is the complicated and progressive system of saving and recovering information that provides regulating, comparing and classifying different instances of the study subject without any kind of loss in content value and diversity between types (Rich 1992). Typologies are generally conceptual and are one of the most famous theory templates (Doty & Glick 1994). Although typology is a mental and innovative process which has no certain approach, Bailey (1994) has designed an approach for typology. In this approach, a full typology can be created based on ideal sample features. This process, which is based on expansion of the ideal type, is called proliferation. In this process, we achieve another set of types using multiplication of dimensions and features of the ideal type and choosing different combinations of them, which is called intermediate or middle types.

Based on the above-mentioned approaches, the first step of this study is to collect disaster management models, which is performed by the library studies and searching of various databases. In the second step of the study, thematic analysis is used for analysing the models. Besides offering the thematic network that has an overarching and almost comprehensive picture of the main elements of disaster management, this stage is a beginning to typology and model classification.

The third step is typology. After analysing the models, basic themes are recognised and are used as theoretical structure to form the types table. By multiplying the theoretical structures by each other and recognising their different combinations, all the possible types are created and named. Then, the models are classified by comparison and adjusting the models to types.

**FIGURE 1:**
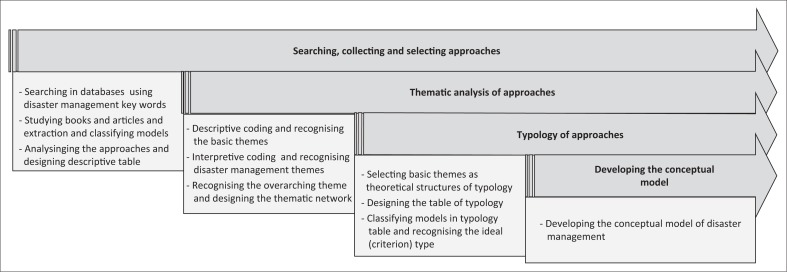
Research process in order to reach a conceptual model of disaster management.

The final step of the study is developing a comprehensive conceptual model for disaster management that is proposed after the analytical discussion on different models and based on the ideal type. Indeed, the distinguished ideal type will be the theoretical credit for researchers’ proposed model. Figure 1 shows the process of research in order to achieve a conceptual model for disaster management.

## Thematic analysis of disaster management models

To find disaster management models, a search based on different combinations of a variety of keywords such as ‘disaster’, ‘disaster management’, ‘disaster management models’, ‘disaster management cycle’, ‘emergency management’, ‘emergency operations plan’, ‘disaster management structure’, ‘emergency management organisation’ and ‘crisis management’ was conducted. A comprehensive literature review of the World Wide Web was performed considering disaster management models. The search was performed in scientific and non-scientific databases because of the nature of research goals. The Web was searched mainly through Google, Bing, Yahoo and Ask search engines. On the other hand, Google Scholar was the main scientific database for investigating scientific materials. A large number of models have been presented by various researchers in this field, most of them for a specific disaster and place. In the first step, more than 100 models were collected in the field of disaster and crisis planning and management. So, after collecting all models, initial analysis was performed and the models that were not general models were excluded from the process of research. By the term ‘general models’, we mean the proposed models in the methodology articles that were written in the conceptual framework of the integrated disaster risk management in the managerial context. Technical notes, case studies, review papers and the other documents which emphasised the technological aspects of the risk analysis were excluded from the database.

Then, considering the main purpose of the study, 38 disaster and crisis planning and management models proposed by different researchers from 1941 to 2016 in different countries were selected for analysis. These models included general models.

Most articles and existing and available documents were studied and there were no comprehensive collection of these models in any book or article. However, Asghar, Alahakoon and Churilov (2006) have categorised disaster management models in four groups. But some of the models that are proposed by combination of different approaches and models are not fitted in any of the proposed groups. Therefore, in this study, another group called combinatorial models is suggested. This group points to a combination of logical, integrated and cause models (Table 1).

**TABLE 1:** Description of disaster management models.

Classification of models	Model title	Modeller (reference)	Abbreviated title	Explanations
Logical models	Traditional model: sequences of action	DPLG-2 (1998)	Traditional model (cycle of disaster management)	The different disaster management phases, rather than in a sequential manner, run parallel to each other, albeit with varying degrees of emphasis.
	Expand and contract model	DPLG-2 (1998)	Expand and contract model	The difference with the traditional model is also often observed that the sequences of action occur simultaneously.
	The four phases model of disaster management	Kimberly (2003)	Kimberly model	This model emphasises emergency management. The most important phase of this model is the response phase.
	The four-stage model of Tuscaloosa	Tuscaloosa (2003)	Tuscaloosa model	This model starts and ends with the response stage.
	Circular model of disaster	Kelly (1998)	Kelly model	The main feature of this model is its ability to learn from real disasters.
	Lechat model	Lechat (1990)	Lechat model	This model starts with anticipation of disaster and ends at the rehabilitation stage.
	The five-stage model of Mitroff and Pearson	Mitroff and Pearson (1993)	Mitroff and Pearson model	This model emphasises the detection and learning phases.
	Gupta stair model	Gupta (2010)	Gupta model	This model does not pay much attention to pre-disaster phases.
	Mitroff model	Mitroff (2000)	Mitroff model	This model is a proactive model that emphasises the learning stage.
	Two-part model of disaster management	Hosseini and Jedi (2006)	Two-part model	This model includes a series of operational and logistic measures. So, this model is called a two-part model.
	Iceberg model	Heinreich (1941)	Iceberg model	The main feature of this model is its attention to the structure and showing seeming template of model.
	Contreras model	Contreras (2016)	Contreras model	In this model, a number of indices have been developed for measuring vulnerability to disasters. The main feature of this model is its attention to the reconstruction after disaster.
Integrated models	Manitoba model	Manitoba Health Disaster Management (2002)	Manitoba model	Advantage and feature of this model is establishing a balance between preparation and resilience, in order to respond to the specific needs of the disaster.
	McConkey linear model	McConkey (1987)	McConkey model	McConkey model pays special attention to pre-disaster management in four stages.
	Weichselgartner integrated model	Weichselgartner (2001)	Weichselgartner model	The overall objectives of this model are the assessment of probable damage and the planning of future measures to reduce this damage.
	Integrated model of Moe and Pathranarakul	Moe and Pathranarakul (2006)	Moe and Pathranarakul model	The results of this model show the importance of proactive and reactive strategies in natural disasters management.
	McEntire et al. integrated model	McEntire et al. (2010)	McEntire et al. model	An integrated approach for modelling the vulnerability should consider social science research, engineering and physics simultaneously.
	Onion model	Mitroff, Shrivastava and Udwadia (1978)	Onion model	This model provides a framework for preparing organisations in the crisis.
	Deming cycle model	Aguayo (1991)	PDCA model	The PDCA cycle with the continuous improvement cycle of plan, do, check and act was advocated after the Second World War.
	Integrated system-oriented model	Meshkati and Tabibzadeh (2016)	Integrated system-oriented model	The main feature of this model is its attention to the emergency response.
	Monitoring and evaluating model of disaster risk management	Scott et al. (2016)	Monitoring and evaluating model of disaster risk management	This model is a unique framework for monitoring and assessment of disaster risk management plans for use by disaster risk management programmes to track the outcomes of their interventions and ultimately raise standards in this area.
Cause models	Crunch cause model	Asian Disaster Preparedness Centre (2000)	Crunch model	This model is a causal model that provides a framework for understanding the causes of the disaster; its structure is formed by the following equation: Disaster Risk = Hazard *Vulnerability.
	Pressure and release (PAR) model	Blaikie, Mainka and McNeely (2005)	PAR model	Unlike the Crunch model and using preventive measures, try to reduce the disaster risk.
	Fink’s comprehensive audit model	Fink (1986)	Fink’s comprehensive audit model	This model determines what events could cause a crisis in each functional area. Once scenarios are developed, action plans should be prepared.
	Littlejohn six-stage model	Littlejohn (1983)	Littlejohn model	This model is a framework that provides basic directives for disaster management.
Combinatorial models	Risk management proactive model	Australian Development Gateway (2008)	Australian Development Gateway model	This model tries to combine logical and integrated model.
	Disaster risk management framework (DRMF) model	Baas et al. (2008)	Baas et al. model	This model has the following three steps: - Risk reduction (Normal) - Emergency response - Recovery.
	Risk management model	BPDMP (2013); Zimmermann and Stössel (2011)	Zimmermann and Kull model	The objective of this model is increment of community resilience and risk reduction using combination of logical and integrated models.
	Wheel-shape disaster management model	Rowshandel Arbatani, Purezzat and Qolipoor (2008)	Wheel-shape model	One of the comprehensive disaster management models is the wheel-shape model that is based on the life cycle of disaster and crisis, as well as its various stages. Also, it is formed by combination of logical and integrated models.
	Cuny comprehensive model	Cuny (1998)	Cuny model	Cuny proposed a cycle for disaster management that is one of the complete cycles. This model considers administrative and management measures that are necessary in disaster management using a combination of logical, integrated and cause models.
	Saldana-Zorrilla model	Saldana-Zorrilla (2015)	Saldana-Zorrilla model	This model provides a set of policy suggestions for integrating risk management and increasing risk reduction measures and planning.
	Institutional model for collaborative disaster risk management	Tau, Niekerk and Becker (2016)	Institutional model for collaborative disaster risk management	This model combines the theoretical, political and technical dimensions of collaboration to enhance buy-in for the disaster risk management and reduction function of governments.
Other models	Ibrahim et al. model	Ibrahim et al. (2003a); Shaluf et al. (2003)	Ibrahim et al. model	This model represents the technological disaster pre-condition stages.
	Gonzalez, Herrero and Pratt model	González, Herrero and Pratt (1996)	González, Herrero and Pratt model	This model states that with the pre-disaster measures, we can change the consequences of the crisis.
	Fink model	Fink (1986); Penrose (2000)	Fink model	This model includes prevention components and crisis analysis.
	Statoil model	Statoil (2013)	Statoil model	This model is a reactive model because it starts the activities after the occurrence of disaster and lasts until returning the condition to the pre-disaster normal condition.
	Pagoda model	Okada (2004)	Pagoda model	City has been considered as a vital five-stage system in this model.
	Octopus model	Shi et al. (2011)	Octopus model	As disasters have complex systems, mutual risk management should be based on multidimensional system for achieving success from policy-making viewpoint. This model is proposed based on this viewpoint.

PAR, Pressure and release; DRMF, Disaster risk management framework; DPLG, Department of Provincial and Local Government; BPDMP, Badakhshan Provincial Disaster Management Plan; PDCA, Plan Do Check Act.

The first category is logical models. Logical models provide a simple definition of disaster stages and emphasise the basic events and actions that constitute a disaster. Traditional model of disaster management is one of the well-known and common logical models. In this model, the traditional process of disaster management has three phases: before, during and after the disaster. The first phase consists of activities such as prevention, mitigation and preparedness, while the second phase includes activities connected to reaction and response and the third phase includes activities such as recovery, reconstruction and development (ADPC 2000).

The second category of models are integrated models. An integrated model of disaster management is a tool for organising the involved activities in order to ensure effective and efficient implementation, and four factors can be identified for it: hazard assessment, risk management, mitigation and preparedness.

The Manitoba model is one of the famous integrated models. This model generally consists of six independent elements, namely strategic plan, hazard assessment, risk management, mitigation, preparedness, and monitoring and evaluation. Each element observes its own boundaries and involves its own set of activities and processes (Manitoba Health Disaster Management 2002). The advantage of this model is that it provides a balance between preparedness and flexibility in order to respond fluidly to the specific needs of disasters. As this model provides the link between actions and events in disasters, such links can be tight or loose.

The third category of these models is cause models. The cause category is not based on the idea of defining stages in a disaster. This category suggests some underlying causes of disasters. The Crunch model is one of them which proposes a frame to understand the causes of a disaster (ADPC 2000; Cannon 2004; Bankoff 2001; Heijmans 2001; Marcus 2005). This model is based on the belief that there are some factors that affect the vulnerability to disasters. In this model, these factors are named as components at risk such as lives and properties of humans, environment and infrastructures. The progression of vulnerability of a community is revealed and the underlying causes that fail to satisfy the demands of the people are identified. The model then goes on to estimate the dynamic pressure and unsafe conditions.

The fourth category consists of combinatorial models in which the logical, integrated and cause models are combined to propose a model. The Cuny model is one of these models which is made by compilation of features of the other three categories (Cuny 1998).

Finally, the fifth category applies to other models in which no features of the other mentioned categories has been used. These models are miscellaneous and refer to a condition that the structure and template of the model is not located in any of the four mentioned categories. For example, Ibrahim, Fakharu’l-razi and Mustapha (2003b) proposed a model to show the pre-condition stages of technological disasters. Details of this model have been raised by Shaluf et al. (2003) and Ibrahim, Fakharu’l-razi and Aini (2003a). This model consists of eight phases: inception of error, accumulation of errors, warning, failure of correction, disaster impending stages, triggering events, emergency stage and disaster.

As this article does not have the capacity of a design and one by one explain models with their schematic views, their general characteristics, including name, reference, modeller, presentation year and conceptual elements of the model, are presented in Table 1. The result of the first step of thematic analysis approach is also presented in this table. According to this table, elements of each model are recognised as the primary themes of the study.

The second step of the study process is analysing the models. In this stage, we attempted to extract and classify themes of each model using thematic analysis. This method requires three stages. In the first stage, which is descriptive coding, existing elements in each model were extracted as code and then the basic themes that are the repetitive and distinguished features in the text were recognised. Then, in the stage of interpretive coding, basic themes were classified in three categories that are called organising themes. These categories are hazard assessment, risk management and management actions. The last stage is determination of the overarching theme which is formed from all the other mentioned themes (King & Horrocks 2010).

### Hazard assessment themes

Hazard assessment means a series of actions including planning and control in order to reduce the consequences of humans’ harmful activities that can cause human casualties and social, economic and environmental damage. Considering the mentioned concept, among the enumerated models for disaster management, some models emphasise the existing factors and the concept of hazard assessment. These factors affect the crisis cycle and performance in different ways. In fact, the basis of good performance can be searched among these items and also the weak performance can be attributed to these factors. Therefore, it can be said that these models explain the reasons and are effective in recognising the reasons of current status and also designing the ideal status because there cannot be any planning or decision-making for development and improvement without identifying the contexts and triggers.

### Risk management themes

The purpose of risk management is generally information assessment (collecting, classifying and analysis) of hazards in order to effectively plan and organise the needed resources for reconstruction and providing a balance in operational power of the city or the organisation after the disaster (Parker 1995). Risk management is, of course, a new approach that has found a good place in the field of crisis and has slowly overcome the traditional approach of crisis management, and in some cases is trying along with it to solve the problems and completing the progress of this subject.

### Management actions themes

Some models have pointed to different stages that they should take continuously for disaster management. In the literature of disaster management, these models are called process-oriented models. Process themes of these models are in a logical sequence and order that separating or prioritising them in terms of frequency will demolish the spirit of the process and the order of stages. Therefore, by continuous comparison of models with each other, hazard assessment, risk management and management actions themes (components) were extracted in terms of frequency (Table 2).

**TABLE 2:** Identified themes in hazard assessment, risk management and management actions.

Component	Number of repetitions
**Hazard assessment**	
Exposure analysis	7
Hazard identification	7
Hazard forecast	6
Hazard analysis	7
Vulnerability assessment	10
Resource assessment	4
**Risk management**	
Risk context	6
Risk communication	2
Risk identification	6
Risk analysis	11
Risk evaluation	6
Risk treatment	14
Monitoring and revising the risk control plan	3
**Management actions**	
Prevention and warning	13
Mitigation	14
Preparedness	17
Response	21
Recovery (reconstruction and rehabilitation)	20
Learning and development	10

### Thematic network

**FIGURE 2:**
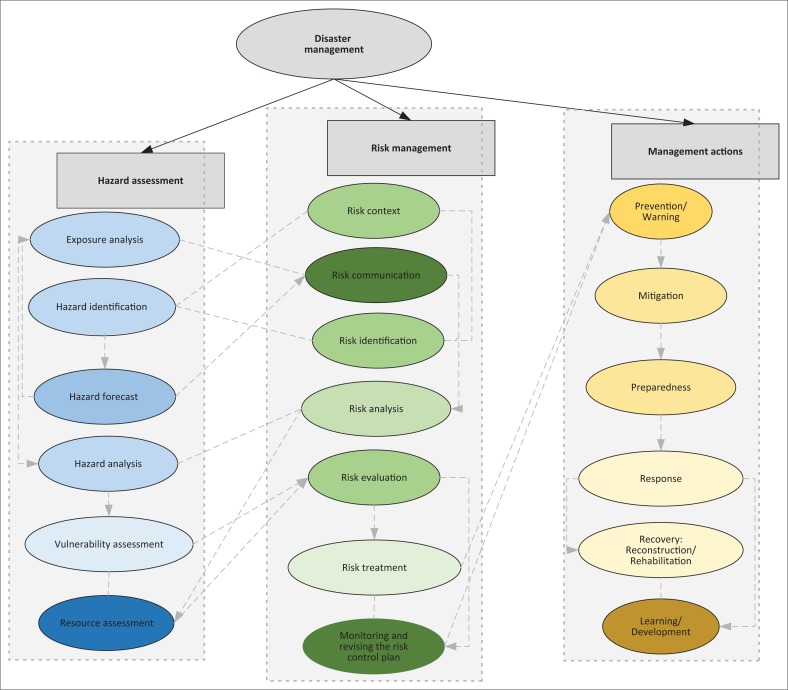
Disaster management thematic network.

One of the thematic analysis tools is drawing a thematic network that simplifies organising of the themes and its purpose is subject perceptions (Attride-Stirling 2001). Based on the basic and organising themes that were obtained from analysis of the models, a thematic network was developed and delineated using interpretive structural modelling (Figure 2). This network shows the connections across columns and between components.

### Models typology

Differences and some variances among different models of disaster management have led to complications in this conceptual structure and theoretical chaos. Bailey (1994) claims that well-formed typology can be very effective in establishing discipline in a chaotic environment and in reducing complication.

Based on the thematic analysis of models, it can be concluded that disaster management models have three main themes; these elements are the three organising themes (i.e. hazard assessment, risk management and management actions). Therefore, a comprehensive model of disaster management should include these themes, which is called the ideal type. Thus, inspired by Bailey’s method in building typology, eight possible types can be recognised from the presence or the absence of each of these themes (Table 3). The most complete type includes all the features which Bailey addresses as the criterion type and the most incomplete one has none of the features. These two types that are placed at two ends of the spectrum are called polar types and other table cells are called middle types (Bailey 1994). These types act as conceptual containers that contain different instances (models).

**TABLE 3:** Typology of disaster management models (first phase).

Hazard assessment	Risk management	Management actions	Status	Typologies	Models
Existence: 1	Existence: 1	Existence: 1	1-1-1	Ideal	The two-part, Manitoba, Weichselgartner, wheel-shape, Cuny and monitoring and evaluating model of disaster risk management models
		Absence: 0	1-1-0	Two-dimensional	McConkey, Crunch, Pressure and Release (PAR), Fink’s Comprehensive Audit, Baas et al. and Fink models
	Absence: 0	Existence: 1	1-0-1	Two-dimensional	Ibrahim et al. model
		Absence: 0	1-0-0	One-dimensional	McEntire et al., Onion, Gonzalez Herrero and Pratt and Pagoda models
Absence: 0	Existence: 1	Existence: 1	0-1-1	Two-dimensional	Mitroff, Moe and Pathranarakul, Australian Development Gateway, Institutional model for collaborative disaster risk management and Saldana-Zorrilla models
		Absence: 0	0-1-0	One-dimensional	PDCA, Littlejohn, Statoil and Octopus models
	Absence: 0	Existence: 1	0-0-1	One-dimensional	Traditional, Expand and contract, Kimberly, Tuscaloosa, Kelly, Lechat, Mitroff and Pearson, Gupta, Iceberg, Zimmermann and Kull, Integrated system-oriented and Contreras models
		Absence: 0	0-0-0	None	-

PDCA, Plan Do Check Act.

Therefore, based on different combinations of the three organising themes, a complete typology was made that includes seven models. According to the presented typology, disaster management models can be classified in three categories: one-dimensional models that include three types of typology table and only pay attention to one theme of comprehensive disaster management, two-dimensional models that form the other three types and have more abundance compared to the one-dimensional models and three-dimensional models that occupy only one type, which is the ideal type.

After surveying the mentioned models, considering the necessity of studying the effective sub-components of disaster management in each identified organising theme, this section discusses sub-components in the ideal type. Table 4 shows the sub-components of three organising themes in the selected ideal models.

**TABLE 4:** Typology of disaster management models (second phase).

Original themes	Components	Selected models					Monitoring and evaluating model of disaster risk management
		The two-part model	Manitoba model	Weichselgartner model	Wheel-shape model	Cuny model	
Hazard assessment	Exposure analysis	✔	-	✔	-	✔	-
	Hazard identification	-	-	-	-	✔	-
	Hazard forecast	✔	-	-	✔	✔	-
	Hazard analysis	-	✔	✔	-	✔	-
	Vulnerability assessment	-	✔	✔	-	✔	✔
	Resource assessment	-	✔	-	✔	✔	-
Risk management	Risk context	✔	-	-	-	-	-
	Risk communication	-	-	-	-	✔	✔
	Risk identification	✔	-	-	✔	✔	-
	Risk analysis	-	✔	✔	-	✔	✔
	Risk evaluation	✔	-	✔	-	✔	-
	Risk treatment	✔	✔	✔	-	✔	-
	Monitoring and revising the risk control plan	-	-	✔	-	-	✔
Management actions	Prevention and warning	✔	-	✔	✔	✔	-
	Mitigation	-	✔	✔	-	✔	-
	Preparedness	-	✔	✔	✔	✔	✔
	Response	✔	-	✔	✔	✔	-
	Recovery (reconstruction and rehabilitation)	✔	-	✔	✔	✔	-
	Learning and development	-	-	-	✔	✔	-

✔ , The studied model has considered the respective component.

Reviewing the mentioned tables, it can be stated that each of the presented models have studied some components in which some of the effective components have been forgotten. Even in studies that have included almost all three organising themes in their models, they have ignored some sub-components of each theme. However, the comprehensive model should include all themes and the related sub-components.

## Results and discussion

As it was mentioned, most of the models have studied some components, while some components have been forgotten. The traditional, Expand and contract, Kimberly, Tuscaloosa, Kelly, Lechat, Mitroff and Pearson, Gupta, Iceberg, Zimmermann and Kull models are examples of one-dimensional type that only paid attention to the managing dimension (factors and indicators). These models can only be used in studying the process of management. Process-oriented models mostly have a logical order and consecutive stages so that imperfect implementation or neglect of each stage will lead the whole process into problems.

Another type of one-dimensional model includes the Plan Do Check Act (PDCA), Littlejohn, Statoil and Octopus models, which only considered risk management. The unique feature of this model is the development and sequence in risk mitigation by focusing on the pre-disaster stage.

The third type of one-dimensional models includes the McEntire et al., Onion, Gonzalez Herrero and Pratt and Pagoda models that pay attention to hazard assessment.

In the two-dimensional type that includes themes such as hazard assessment and management actions, the only model is the Ibrahim et al. model. Indeed, this model consists of two parts, one within the other. The first stage from the eight-stage pattern of Ibrahim et al. is determination of consequences that will end eventually with occurrence of an emergency and disaster condition. This model is proposed to show the pre-condition stages of technological disasters. In this study, the eight-stage model has been combined with a three-stage pattern of the study and is mentioned as a two-dimensional model.

The two-dimensional type that includes the themes of risk management and management actions includes models such as the Mitroff, Moe and Pathranarakul and Australian Development Gateway models. These models can be called operational management models because they only pay attention to process and the way of management without considering hazard assessment. For example, in the Mitroff model, the operational aspect is considered and its structure is proposed through risk management. The special feature of this model is that it is a proactive model that pays much attention to the learning element.

In the McConkey, Crunch, Pressure and Release (PAR), Fink’s Comprehensive Audit, Baas et al. and Fink models, there has been a great deal of attention to hazard assessment and risk management. In these models, the only missing link among the three main study components is the process or management actions. So, these models are also included in the two-dimensional models category.

Finally, the seventh type, which is called the ideal (criterion) type, includes the three-dimensional models. These models can be called comprehensive models of disaster management because they include all three organising themes. Although the two-part model of crisis management is a processing and operational model in an overview by reviewing factors such as prevention, response and reconstruction, but in the first stage of process that is recognising the hazard with an emphasis on exposure analysis, it is a multidimensional model and has studied risk management dimensions (i.e. risk context, identify risk, evaluate and treat risk) at the end.

The Manitoba model initially proposes a three-stage model that constantly tries to aggregate the components of comprehensive disaster management. By reviewing the three organising themes, this model tries to propose an appropriate model. The feature and advantage of this model is providing balance between preparedness and flexibility (resilience) in order to respond to the disasters, special needs.

The main purpose of the Weichselgartner model is assessment of probable damages and planning for future actions to reduce these damages. This model also tries to propose a comprehensive model by studying components that are shown in Table 4.

The wheel-shape model is also considered a processing-operational model, but it can be considered as one of the three options of models by reviewing components such as resource assessment in the field of hazard assessment and risk identification in the field of risk management.

But among all the mentioned models in this study, only the Cuny model has reviewed the components more comprehensively and has covered most of the study’s desired components, except risk context and monitoring in the field of risk management. Cuny has proposed a cycle for disaster management that considers all the required management and executive actions that need to be carried out in the course of a disaster. In this cycle, there are several stages and sub-stages so that deliniating boundaries is not possible among them. Besides, sometimes, according to the disaster type, transposition of these stages change and sometimes, a number of these stages do not exist among some models. In other words, a combination of logical, integrated and cause models has been used in this model’s structure.

**FIGURE 3:**
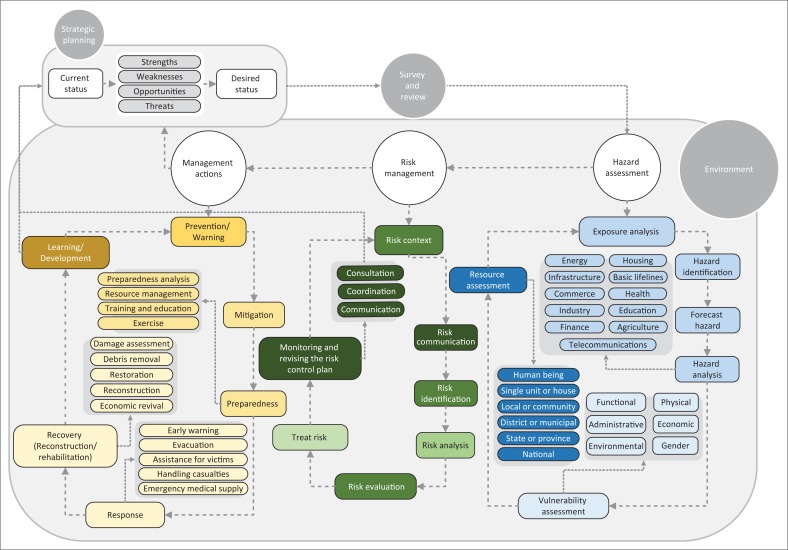
Comprehensive conceptual model of disaster management.

Nevertheless, it becomes clear that some scientists have looked at disaster management one-dimensionally; even in some two-dimensional models, one dimension has more advantage. While the proposed typology in this study indicated that based on the ideal type, the comprehensive model should include three above-mentioned themes. Considering this point and by studying the mentioned models, the proposed conceptual model of this study is presented in Figure 3. This model and three stages of process and concepts within them, relations and their transposition are the results of thematic analysis of the literature review.

Based on this conceptual model for developing a comprehensive model of disaster management, initially three main questions should be answered:

- What is the role of environmental factors in this model?
- How are the elements that received the least attention in the previous models covered in the proposed model?
- How is the position of feedback exerted and applied in this study?

As disasters’ dimensions are different in various places, each organisation should determine their performance level and dimensions in accordance with the requirements and based on their vision, mission, goals and strategies and then try to design the indicators. So, every organisation or part should act at various levels in disaster management considering existing resources and try to reduce the consequences of disaster in different spaces with the consideration of vulnerability factors such as social and economic factors. Therefore, based on the performed study and the above-mentioned model, the environmental factor is an inseparable factor in different parts of the model and it should be checked on all three levels.

Various models have proposed an approach for disaster management, so their role is significant in previous disasters. Now that special attention of each model to a special context has prevented them from paying attention to all effective factors in management. Based on this point, in the current study, we attempted to complete the previous models and, considering the colour spectrum, proposed in the model. The issues that have not been considered in the previous models are shown with dark surfaces.

In the proposed model, feedback’s position is determined using measures such as consultation, coordination and communication in order to complete the previous models. In most proposed models so far, this point was forgotten as a missing link and few models have considered it as an effective option in the field of disaster.

The final point about the proposed model is that hazard assessment is a prerequisite element for risk management, not a part of it. If vision, mission, objectives and strategies have been pointed out in the model’s first stage, the intention is not just writing about them but to study and contemplate them in order to determine effective dimensions and factors of comprehensive disaster management. Therefore, in disaster management, goals and strategies that are written in hazard assessment, risk management and management actions are used to determine the key factors and dimensions and performance measures.

## Conclusion

In this study, a comprehensive conceptual model for disaster management was presented. To overcome the complications and conceptual inconsistencies among the models, thematic analysis, classification and typology were used. In this regard, first, the models of disaster management were collected. In the next stage, the themes of each model were extracted and categorised using thematic analysis in three phases. In the first phase, available elements in each model were extracted as code and the basic themes were recognised. Then, in the phase of interpretive coding, basic themes were classified in three categories that are called organising themes. Finally, in the last phase, a global or overarching theme that consists of all the other mentioned themes was determined. Basic themes which were obtained during interpretive coding are the themes of hazard assessment, risk management and management actions. Based on thematic analysis of the models, it can be concluded that disaster management has three main elements, namely the three organising themes. Therefore, the comprehensive model of disaster management should include these three themes and their sub-basic themes that is called the ideal type that includes all the features and is also known as the criterion type. Results showed that some scientists have looked at disaster management one-dimensionally (one theme). Even in two-dimensional models, one dimension has advantage over the other one. Special attention of each model to a special context has prevented them from paying attention to all the effective factors in efficient and comprehensive management and the role of environmental factors is ignored in most of the models. However, the proposed typology in this study showed that, considering the ideal type, the comprehensive model should include all the three mentioned themes.

Finally, considering this point, the suggested conceptual model of this study was proposed, using the concepts of strategic and comprehensive management. According to the proposed conceptual model, the strategic plan of disaster management should be performed under a comprehensive management, considering all the aspects which are connected with disasters and using the comprehensive management pattern. Also, in the proposed model, feedback’s position was determined using measures such as consultation, coordination and communication in order to complete the previous proposed models. In most proposed models so far, this point was forgotten as a missing link and few models have considered it as an effective option in the field of disaster. Finally, the proposed model may be applied in the various phases of disasters in different places.
